# Genome-wide association analysis of milk yield traits in Nordic Red Cattle using imputed whole genome sequence variants

**DOI:** 10.1186/s12863-016-0363-8

**Published:** 2016-03-22

**Authors:** T. Iso-Touru, G. Sahana, B. Guldbrandtsen, M. S. Lund, J. Vilkki

**Affiliations:** Animal Genomics, Green Technology, Natural Resources Institute Finland (Luke), Jokioinen, Finland; Center for Quantitative Genetics and Genomics, Department of Molecular Biology and Genetics, Aarhus University, Tjele, Denmark

**Keywords:** Milk traits, Nordic Red Cattle, Whole genome sequence, Association study

## Abstract

**Background:**

The Nordic Red Cattle consisting of three different populations from Finland, Sweden and Denmark are under a joint breeding value estimation system. The long history of recording of production and health traits offers a great opportunity to study production traits and identify causal variants behind them. In this study, we used whole genome sequence level data from 4280 progeny tested Nordic Red Cattle bulls to scan the genome for loci affecting milk, fat and protein yields.

**Results:**

Using a genome-wise significance threshold, regions on *Bos taurus* chromosomes 5, 14, 23, 25 and 26 were associated with fat yield. Regions on chromosomes 5, 14, 16, 19, 20 and 25 were associated with milk yield and chromosomes 5, 14 and 25 had regions associated with protein yield. Significantly associated variations were found in 227 genes for fat yield, 72 genes for milk yield and 30 genes for protein yield. Ingenuity Pathway Analysis was used to identify networks connecting these genes displaying significant hits. When compared to previously mapped genomic regions associated with fertility, significantly associated variations were found in 5 genes common for fat yield and fertility, thus linking these two traits via biological networks.

**Conclusion:**

This is the first time when whole genome sequence data is utilized to study genomic regions affecting milk production in the Nordic Red Cattle population. Sequence level data offers the possibility to study quantitative traits in detail but still cannot unambiguously reveal which of the associated variations is causative. Linkage disequilibrium creates difficulties to pinpoint the causative genes and variations. One solution to overcome these difficulties is the identification of the functional gene networks and pathways to reveal important interacting genes as candidates for the observed effects. This information on target genomic regions may be exploited to improve genomic prediction.

**Electronic supplementary material:**

The online version of this article (doi:10.1186/s12863-016-0363-8) contains supplementary material, which is available to authorized users.

## Background

The number of dairy cows in the Nordic countries has been decreasing during the 21^st^ century [1]. However, total milk production levels have remained stable, as milk yield per cow has increased. For example in Finland (including all dairy breeds) the average production per cow per year has increased from 6786 l (2000) to 8201 l (2014), while fat and milk contents have remained fairly constant [2].

Global yearly milk consumption per capita is increasing, and global demand for animal based foods is expected to be doubled by 2050 [3], driven by both population growth and increased consumer preferences for meat and milk products. Ruminants are unique in their capacity to digest fibre and convert non-edible resources into high quality human nutrition, making them highly relevant for meeting the increasing global demand for food. While animal breeders have achieved considerable improvements in production traits, cow fertility has been declining [4–6]. However, during the recent years, the decrease in cow fertility in Nordic countries has been slowing down and even is refracted, due to the weighting of fertility traits in the breeding program [7]. Many female fertility traits in dairy cattle show antagonistic genetic correlations with milk production traits [8] but with low or moderate correlations [9]. This implies that simultaneous genetic selection for increased milk yield and reproductive performance is possible [9]. Simultaneous breeding for both productive and fertile cows would benefit substantially from knowing the genetic and physiological links between production and health to disentangle the effects on these traits. Recent results in Holstein and Jersey breeds indicate little or no overlap between genomic regions associated with milk yield and fertility [10, 11].

Genome wide association studies (GWAS) have benefited from the rapid development of single nucleotide polymorphism (SNP) genotyping technologies, but despite of the relatively high density of the available SNP chips, finding the causative mutation is not straightforward. The high level of linkage disequilibrium in dairy cattle results in long quantitative trait loci (QTL) regions with several possible candidate genes. Using whole genome level sequence variants for association analyses would be an ultimate choice, because then the causative variant is most likely included among the studied variants. Potentially this helps to pinpoint the causative mutations thus leading to a better understanding of biological mechanisms behind the QTL [12] and improve the efficiency of genomic selection [13]. Using sequence level SNPs will also enable identification of SNPs that explain a small fraction of the trait variation because either the causal SNP and/or SNP(s) with high linkage disequilibrium (LD) with the causal variant are included in the analysis [13].

Historically separated three dairy breeds Finnish Ayrshire from Finland, Danish Red from Denmark and Swedish Red from Sweden are at present under a joint breeding value estimation system, known as the Nordic Cattle Genetic Evaluation [14]. Previous QTL studies of milk traits in Nordic Red Cattle (NRC) have been done within the subpopulations with microsatellite markers and fairly small sample sizes (e.g. [15, 16]). The objective of this study was to use variations at the genome sequence level to carry out association study for milk, fat and protein yields in NRC; to identify potential causal variants and understand the genetic architecture of these traits. In addition, the data provides the possibility to compare the results to similar studies for fertility traits in the NRC [17], to reveal potential QTL with antagonistic effects for milk production and fertility traits.

## Methods

No animal experiments were performed in this study, and, therefore, approval from the ethics committee was not required. Semen samples were collected for breeding purposes by local organizations with appropriate permits.

Milk, fat and protein yields’ trait definitions are standardized across the Nordic countries. Phenotypic records for dairy cattle are housed in a centralized database [14]. Breeding values for milk, fat and protein yield (MY, FY and PY) are based on production figures expressed in kilograms taken from routine milk records and then combined into an index for each trait. For details on genetic evaluation for milk yield traits in Nordic countries see [18]. The breeding values used for association analysis were de-regressed breeding values from the routine genetic evaluation by NAV (Nordic cattle genetic evaluation) and were available for 4280 progeny tested NRC bulls (2127 from Finland, 1217 from Sweden, 915 from Denmark and 21 from other countries). The reliabilities of the deregressed breeding values were in the range of 0.67 to 0.99 with a mean of 0.95 and the first quartile at 0.94.

### SNP array genotyping

All 4280 NRC bulls with deregressed breeding values were genotyped using *BovineSNP50 BeadChip* SNP array version 1 or 2 (Illumina Inc., San Diego, CA). DNA was extracted using standard procedures from semen samples. Chip typings were done by GenoSkan A/S, Tjele, Denmark or labs belonging to Aarhus University. The quality parameters used for selection of SNPs were minimum call rates of 85 % for individuals and 95 % for loci. Marker loci with minor allele frequencies below 5 % and deviation from Hardy-Weinberg proportion (*P* < 0.00001) were excluded. The minimal acceptable GC score was 0.60 for individual typings, and individuals with average GC scores below 0.65 were excluded. The number of SNP remaining after quality control was 43,415 in the genotypes obtained from *BovineSNP50 BeadChip* SNP array (50 K data set). The genome positions of the SNPs were according to the UMD3.1 Bovine genome assembly [19].

### Imputation to whole genome sequences

The 50 K genotypes of these bulls were imputed to whole genome sequence data using a two-step approach [20]. Genotypes from 50 K chip for each bull were first imputed to a high-density SNP array (HD) using a multi-breed reference of 3383 animals (1222 Holstein, 1326 NRC and 835 Danish Jersey individuals) which had been genotyped with the Illumina BovineHD chip (Illumina Inc., San Diego, CA). The number of SNPs, after imputation to the BovineHD chip, was 648,219. These imputed HD genotypes were subsequently imputed to the whole genome sequence level using a multi-breed reference panel of 1228 animals from *Run4* of the 1000 Bull Genomes Project [13, 21] and additional whole genome sequences from Aarhus University [22] including 368 Holstein, 86 RDC, 88 Jersey and rest from number of cattle breeds. Datasets (SNP array types and whole sequence) were pre-phased with BEAGLE4 r1274 [23] and genotype imputation were done using *Minimac2* [24]. The imputation accuracy for this data is reported earlier [25], but with a smaller whole genome sequence reference population. Sequence variants having imputation accuracy r^2^ (ratio of empirically observed variance of the allele dosages to the expected binomial variance at Hardy-Weinberg equilibrium and was obtained from *Minimac2* software output) less than 0.5 were filtered away. The mean accuracy for the variants with r^2^ > 0.5 was 0.94.

### Association analysis

The association analysis for each of the imputed sequence variants (minor allele frequency, MAF > 0.005 and deviation from Hardy-Weinberg proportion > 0.00001) was carried out using a two-step variance components-based approach to account for population stratification implemented in the EMMAX software tool [26]. In a first step, the polygenic and error variances are estimated using following variance component model:

$$ \boldsymbol{y}=1\boldsymbol{\mu} +\boldsymbol{a}+\boldsymbol{e} $$

where ***y*** is a vector of de-regressed breeding values, **1** is a vector of ones, μ is the intercept, ***G*** is the kinship matrix built based on high-density SNP genotypes using EMMAX software, ***a*** is a vector of breeding values assumed to have a multivariate normal distribution ***a*** ~ N(**0**, ***G****σ*_*a*_^2^), ***e*** is a vector of random residuals assumed to have a multivariate normal distribution ***e*** ~ N(**0**, ***I****σ*_*e*_^2^), where ***I*** is an identity matrix, *σ*_*a*_^2^ is the additive genetic variance and *σ*_*e*_^2^ is the error variance.

In a second step, the SNP effect is obtained using a linear regression model:

$$ \boldsymbol{y}=\mathbf{1}\boldsymbol{\mu } + \boldsymbol{x}\boldsymbol{b} + \boldsymbol{\eta}, $$

where **x** is a vector of imputed genotype dosages (ranged between 0 and 2), *b* is the allele substitution effect and **η** is a vector of random residual deviates with (co)variance structure *Gσ*_*a*_^2^ + *Iσ*_*e*_^2^.

### Search for multiple QTL in a genomic region

To test if multiple QTL are segregating in a genomic region we included the most significant or the known causal variant as cofactor in the model and check for additional QTL in a genomic region for fat yield on chromosomes 14, 25 and 26 and for milk yield on chromosome 14. We fitted the SNPs (Additional file 1) as fixed effect to a linear mixed model.

The statistical model is described by the formula:

$$ \boldsymbol{y}=\mathbf{1}\mu +\boldsymbol{q}sn{p}_{top}+\boldsymbol{x}g+\boldsymbol{Z}\boldsymbol{u}+\boldsymbol{e} $$

where **y**, **1**, *μ* are described as in the EMMAX model, *snp*_*top*_ is the effect of the SNP fitted as co-factor in the model, and *g* is the additive genetic effect of the the SNP under study, ***q*** and **x** are vectors of SNP genotype dosages (ranging from 0 to 2), and **u** is a vector of random polygenic effects, which are normally distributed **u** ~ N(0, **A***σ*_*u*_^2^), where **A** is the pedigree-based additive relationship matrix, *σ*_*u*_^2^ is the polygenic variance, **Z** is an incidence matrix relating phenotypes to the corresponding random polygenic effects, and **e** is a vector of residual effects, which are normally distributed **e** ~ N(0, **D***σ*_*e*_^2^), where **D** is a diagonal matrix with elements *d*_*ii*_ = (1 − *r*_*DRP*_^2^)/*r*_*DRP*_^2^ to account for heterogeneous residual variances due to different reliabilities of DRP (*r*_*DRP*_^2^), and *σ*_*e*_^2^ is the residual variance. Analyses were performed using the DMU package [27]. Significance testing of SNP effects was performed using a two-sided t-test. The null hypothesis was g = 0. After that, the Bonferroni correction was applied same as in the EMMAX analysis to control for false positive associations.

The genome-wise significance threshold corresponding to an error rate of 0.05 was set at 3.16 × 10^−9^ after correction for multiple testing using a Bonferroni correction of 15,679,852, 15,679,853 and 15,679,844 independent tests for fat, milk and protein yield respectively. Only SNPs with the *p* value less than 3.16 × 10^−9^ (−log_10_(*p*) ≥ 8.50) were annotated with the variant effect predictor (VEP) tool using the Ensembl database, Release 82 [28]. The prediction whether an amino acid substitution caused by missense variation affects protein function was estimated by SIFT analysis [29] implemented in VEP tool [28]. The SIFT prediction is based on sequence homology and the physical properties of amino acids. Manhattan plots were created with the qqman v.0.1.2 R package [30]. In addition, we compared our findings to results obtained from study by Höglund et al. [17] where a similar genome-wide association study for female fertility in Nordic Red cattle was conducted.

### Ingenuity pathway analysis and enrichment analysis

Lists of genes with significant hits from the QTL peak regions associated with each milk trait were uploaded into the Qiagen’s Ingenuity® Pathway Analysis IPA® [31]. For this purpose, also SNPs significantly associated with Fertility index (FI) in the Nordic Red Cattle [17] were annotated with the VEP tool [28] and genes having one or more significant SNP were analyzed with IPA® [31].

Biomart tool [32] embedded in Ensembl database [33], was used for searching human homologs for cow Ensembl IDs for the genes. In case there was more than one, all reported human orthologs were kept. The human homologue lists for each trait included 214, 69, 66 and 263 genes for fat, milk, protein yields and fertility index, respectively. After running core analysis for each trait, networks based on the information of gene connectivity in Ingenuity Knowledge Database with highest score-values were considered. Score-value represents the negative log of the *p*-value for the likelihood that the molecules would be found together by chance.

Gene ontology (GO) term enrichment analysis with genes found within the top SNPs was performed with a Singular Enrichment Analysis (SEA, Fisher’s exact test, FDR < 0.01) provided by AgriGO webpage [34].

## Results

Depending on the trait we could identify several thousands (3594, FY), less than a thousand (755, MY) or less than a hundred (85, PY) significantly associated SNPs (Bonferroni corrected threshold for significance -log_10_(*p*) ≥ 8.50; Additional files 2, 3 and 4). No common significantly associated SNPs were found between this study and with those found for female fertility traits [17]. However, significantly associated SNPs were found in five common genes between fertility and fat yield. These five genes are located on chromosomes 25 (*ENSBTAG00000034643*) and 26 (*GBF1*, *TMEM180*, *ACTR1B*, and *bta-mir-146b*).

The summary of the annotations for significantly associated SNPs are presented in Table 1. Among annotated SNPs, intron variants are the most common type for each of the traits. SNPs changing amino acid (missense variations) are rare, 21 for FY, 1 for MY, 3 for PY and 18 shared between FY and MY. Seven missense variations were predicted by SIFT analysis [29] to be deleterious, i.e. potentially leading to changes in the function of the protein (Additional file 5). Splice region variants are rarer forming only one percent or less from the total amount of significantly associated SNPs (Additional file 5).

**Table 1:** The number of significant SNPs (- log_10_(*p*) ≥8.50) for each trait and how SNPs are divided into different consequences. SNPs were annotated with the variant effect predictor –tool [28]. One SNP can have more than one annotation

Trait	Number of significant SNPs	Intron variant	Intergenic variant	Downstream gene variant	Upstream gene variant	Synonymous variant	Missense variant	3′ UTR variant	Splice region variant, intron variant	5′ UTR variant	Splice region variant, synonymous variant
Fat yield	3594	1641	1307	600	551	94	40	39	9	6	2
Milk yield	755	322	195	301	240	42	20	16	5	1	2
Prot. yield	85	50	14	32	12	3	3	1	1		

Results for fat yield within the larger associated areas on chromosomes 14, 25 and 26 were further examined by fixing the effect of top SNP(s). Only peaks that remained after fixing the other top SNPs were considered as potential QTL in these chromosomes.

**Fig. 1:**
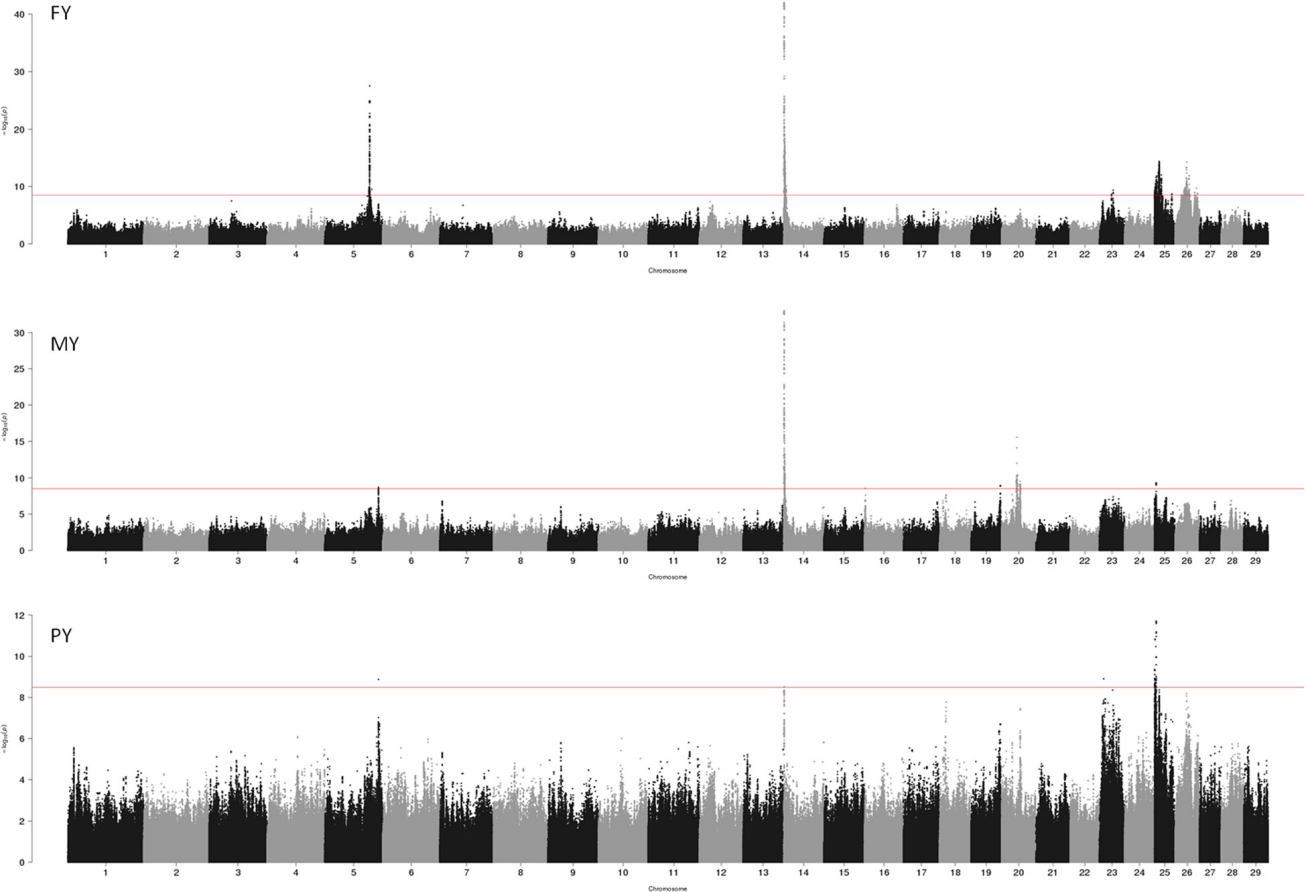
Genome-wide Manhattan plots for the fat yield (FY), milk yield (MY) and protein yield (PY). Red line indicates the genome-wide significance level (−log_10_(*p*) = 8.50)

Seven, eight and four separate QTL regions (“peaks”) were defined for fat, milk and protein yield, respectively (Fig. 1, Additional files 6, 7, 8, 9, 10, 11, 12 and 13). The peaks were defined as continuous regions containing SNPs having –log_10_(*p*) ≥ 8.50. Top SNPs with the consequences for each defined QTL region per trait are listed in Table 2.

**Table 2:** QTL regions for each of the trait. Top SNP for each QTL are shown including position, -log_10_(*p*)-values, minor allele frequency (MAF), gene information, annotation of the top SNP, allele substitution effect (b.value) and standard error for b.value (SE)

CHR	Start (bp)	End (bp)	Length of the QTL region (bp)	Significant SNPs in region	No of genes with significant SNPs within the QTL region	Top SNP	Position of the top SNP (bp)	- log_10_(*p*)	MAF	Gene	Annotation of the top SNP	b.value	SE
Fat yield													
5	92,372,732	94,425,668	2,052,936	330	3	rs209818856	93,945,694	27.49	0.38	*MGST1*	intron variant	−2.807	0.253
14	1,448,510	2271832	823,322	509	49	rs136783505	1,807,140	42.01	0.07	*DGAT1/HSF1*	downstream variant/intron variant	−6.709	0.484
23	28,567,796	28,591,530	23,734	5	1	rs381390819	28,567,796	9.36	0.45	*TRIM26*	intron variant	1.148	0.184
25	8,222,347	11,507,986	3,285,639	883	16	rs379546164	9,870,005	14.40	0.24	*CLEC16A*	intron variant	−1.769	0.224
25	36,226,978	36,227,132	154	2		rs109480808	36,226,978	8.72	0.16		intergenic variant	1.635	0.272
26	22,144,777	24,793,744	2,648,967	595	32	rs438420348	24,379,571	14.28	0.16	*NEURL1*	intron variant	−2.273	0.290
26	44,802,991	44,802,991	0	1		rs135624939	44,802,991	9.72	0.28		intergenic variant	−1.474	0.231
Milk yield													
5	112,343,204	11,2450,860	107,656	2	1	rs383553819	112,343,204	8.70	0.36	*MKL1*	intron variant	1.437	0.239
14	1,448,510	2,271,832	823,322	455	48	rs133033480	1,743,939	33.01	0.09	*CPSF1/ADCK5*	downstream variant/splice region variant, intron variant	6.266	0.513
16	1,322,611	1,322,611	0	1	1	rs108979795	1,322,611	8.58	0.28	*LAX1*	upstream variant	−1.451	0.243
19	61,447,138	61,449,096	1958	5		rs210324693	61,449,096	8.93	0.32		intergenic variant	1.490	0.244
20	30,531,217	32,952,019	2,420,802	74	5	rs385640152	31,909,478	15.56	0.11	*GHR*	missense variant	−3.877	0.472
20	37,766,226	39,183,141	1,416,915	34	3	NA	38,828,254	9.54	0.16		intergenic variant	−2.250	0.356
25	2,669,704	2,669,704	0	1		rs209691835	2,669,704	9.21	0.13		intergenic variant	−2.855	0.460
25	3,494,706	3,516,671	21,965	13	4	rs110749311	3,498,960	9.32	0.41	*PAM16/GLIS2*	downstream variant	1.225	0.196
Protein yield													
5	112,450,860	112,450,860	0	1		rs109041054	112,450,860	8.88	0.48		intergenic variant	−1.473	0.242
14	1,802,667	1,802,667	0	1	2	NA	1,802,667	8.52	0.06	*DGAT1/HSF1*	intron variant/downstream variant	3.354	0.564
25	1,094,996	1,257,612	162,616	12	3	rs136085792	1,103,856	10.83	0.22	*UNKL*	intron variant	1.694	0.250
25	3,306,363	3,516,671	210,308	40	8	rs110749311	3,498,960	11.70	0.41	*PAM16/GLIS2*	downstream variant	1.427	0.202

The highest peak was observed on *Bos taurus* chromosome 14 (BTA14) (Additional file 7) spanning the region from 1,448,510 bp to 2,271,832 bp for fat yield (509 SNPs having –log_10_(*p*) ≥ 8.50) and from 1,448,510 bp to 2,271,832 bp for milk yield (455 SNPs having –log_10_(*p*) ≥ 8.50). The highest –log_10_(*p*) values within these regions were obtained for SNPs rs136783505 (bp 1,807,140) for fat yield and rs133033480 (bp 1,743,939) for milk yield. The highest peak for protein yield was located on BTA25 (Additional file 11) within the region 3,306,363–3,516,671 which contained 40 SNPs, the highest *p* value being for SNP rs110749311 (bp 3,498,960).

### Fat yield

We identified seven different QTL regions on five different chromosomes affecting fat yield (Table 2, Fig. 1, Additional file 2). The strongest association found for fat yield was located on BTA14 (Additional file 7) in the *DGAT1* (Diacylglycerol O-acyltransferase 1) gene region. In our data the strongest association is for the variation rs136783505 (bp 1,807,140) with no functional annotation, located 2578 bp downstream of the *DGAT1* gene (Table 2). However, several other variants located nearby, including the previously identified causative variant *K232A* [35] at bp 1,802,266, show similarly high -log_10_(*p*) (Additional file 2). To investigate the significance of other SNPs in the region, we fitted the variation *K232A* as fixed effect. None of the other SNPs remained significant (Additional file 14) after the fixation of the *K232A* variation.

Other SNPs with strong associations with fat yield were found on BTA5 (Additional file 6), BTA23 (Additional file 11), BTA25 (Additional file 12) and BTA26 (Additional file 13).

The QTL region on BTA5 is located between 92,372,732 bp and 94,425,668 bp and the variation with the strongest association (rs209818856, pos. 93,945,694) is located in an intron of the gene *MGST1* (Table 2). On BTA23 the association signal for fat yield comes from the region 28,567,796–28,591,530, the top variation being located at bp 28,567,796 in the intron of gene *TRIM26* (Table 2).

On BTA25 and BTA26, complex patterns of association were seen (Additional files 12 and 13). To clarify the number of independent QTL within these regions we investigated the significance of the SNPs by fitting the top SNPs (five for BTA25 and eight for BTA26, Additional file 1) as fixed effects alone and in different combinations. On BTA 25, significant associations remained at bp 9,870,005 (intronic region of the *CLEC16A* gene) and at bp 36,226,978 (intergenic region) (Table 2). Two QTL remained also on BTA26, one in the region of the *NEURL1* gene (position 24,379,571) and the other in an intergenic region (top SNP at bp 44,802,991) (Table 2).

### Milk yield

In all, eight QTL regions were found for milk yield (Table 2, Fig. 1, Additional file 3). They were located on six different chromosomes (BTA5, BTA14, BTA16, BTA19, BTA20 and BTA25). The strongest association with milk yield was found on BTA14 (1,448,510–2,271,832) having top variation located at bp 1,743,939. This variation is within two overlapping genes, *CPSF1* and *ADCK5*. The QTL region is the same as was found associated with fat yield but with different top variation. As for fat yield, the significance of other than the previously known causative *DGAT1* variation [35] was tested by fitting the variation *K232A* as a fixed effect. None of the other SNPs remained significant after fixing the *K232A* effect (Additional file 15).

The weakest significant association was located on BTA5 with top variation rs383553819 (position 112,343,204) located in an intron of the gene *MKL1* (Additional file 6). On BTA19 the associated region contained no annotated genes (61,447,138–61,449,096), and the top variation rs210324693 (bp 61,449,096 bp) was located in an intergenic region (Table 2, Additional file 9). Two QTL regions were detected on BTA20 (Additional file 10). The QTL were located in the regions 30,531,217–32,952,019 and 37,766,226–39,183,141. The known causative variation *F279Y* (bp. 31,909,478) for milk traits in the gene *GHR* [36] was the top SNP in our analysis for the QTL located in the first region. The top variation within the second QTL was located at bp 38,828,254 in the intergenic region, but this QTL region also includes the *PRLR* gene previously indicated to be linked to milk production (e.g. [37]). BTA25 harbors two QTL, the first at bp 2,669,704 and the second in the region from 3,494,706 bp to 3,516,671 bp, the top SNP located at bp 3,498,960 downstream from the gene *PAM16* (Additional file 12).

### Protein yield

In all, protein yield did not show as many significantly associated SNPs as observed for fat and milk yield (Table 2, Fig. 1, Additional file 4). Chromosomes having QTL for protein yield were BTA5, BTA14, and BTA25. For both BTA5 and BTA14, only one variant for each reached the significance cut-off level; on BTA5 an intergenic variation at bp 112,450,860 (Additional file 6) close to the variant found for milk yield and on BTA14, the variation (bp 1,802,667) located in an intron of the *DGAT1* gene (Additional file 7).

The two other QTL for protein yield were both located on BTA25. The QTL on BTA25 at 3,306,363–3,516,671 overlapped with the QTL found for milk yield and displayed the same top SNP (bp 3,498,960). The other QTL on BTA25 was unique for protein yield, top SNP (bp 1,103,856) located in the gene *UNKL.*

### Networks of associated genes and enrichment analysis

The networks with highest scores for each trait are presented in Additional files 16, 17 and 18. The two top networks from genes associated with fat yield (Additional file 16) had scores of 49, the network associated with carbohydrate metabolism, gene expression and lipid metabolism is presented in Additional file 16a. Additional file 16b shows the network generated from the genes associated to fertility index. The score value for this network is 41 and it consists of genes associated with inflammatory response, cell-to-cell signaling and lymphoid tissue structure and development. Even though common significantly associated SNPs were not found between this study and that of [17] on female fertility traits, significantly associated SNPs were found in five common genes. Two of them (*GBF1* and *bta-mir-146b)* are present in both fat and fertility networks (Additional file 16a and b). The top network for milk yield (score 43, Additional file 17) was associated with functions molecular transport, organ morphology and organismal development. It includes the known milk candidate genes *DGAT1*, *GHR* and *PRLR*, as well as the top hits from BTA5 (*MLK1*) and BTA16 (*LAX1*). The top network for protein yield (score 35, Additional file 18) is connected to functions cell death and survival, cancer and organismal injury.

Altogether 18, 18, 11 and 16 GO terms were significantly (false discovery rate, FDR < 0.01) enriched for FY, MY, PY and FI, respectively. A broad GO term, multicellular organismal process, was the most significant for all four traits, totally eight terms were shared between them. For example all traits were having QTL in the regions containing significant enrichment of genes related to reproduction and reproductive processes (Table 3).

**Table 3:** GO enrichment terms having FDR < 0.01 from the genes having significant variations (−log_10_(*p*) ≥8.50) for fat yield (FY), milk yield (MY), protein yield (PY) and fertility index (FI)

		FY	MY	PY	FI
GO term	Description	FDR	FDR	FDR	FDR
GO:0022610	Biological adhesion	6.20E-07	0.0013		2.00E-06
GO:0065007	Biological regulation	9.40E-12	6.80E-05	0.072	2.40E-28
GO:0044085	Cellular component biogenesis	3.60E-09	0.048		1.00E-22
GO:0016043	Cellular component organization	1.00E-37	2.90E-12	0.00027	1.80E-76
GO:0009987	Cellular process	4.90E-14	4.60E-05	0.0074	3.80E-20
GO:0016265	Death	7.80E-21	2.00E-07		4.00E-25
GO:0032502	Developmental process	8.80E-102	2.10E-41	1.40E-14	
GO:0051234	Establishment of localization	1.80E-14	2.80E-05	0.0057	1.70E-27
GO:0040007	Growth	5.60E-42	2.10E-17		9.40E-45
GO:0002376	Immune system process	0.00022	0.0032		9.80E-10
GO:0051179	Localization	5.90E-20	1.10E-07	0.0059	4.70E-44
GO:0008152	Metabolic process	9.40E-12	0.00035	0.57	7.80E-18
GO:0032501	Multicellular organismal process	8.00E-126	1.10E-44	1.50E-20	1.20E-161
GO:0048519	Negative regulation of biological process	2.80E-50	1.30E-25	1.20E-07	
GO:0048518	Positive regulation of biological process		1.70E-32	1.60E-08	
GO:0050789	Regulation of biological process	7.10E-10	0.00011	0.32	6.90E-22
GO:0000003	Reproduction	3.70E-57	3.70E-14	3.50E-09	1.20E-54
GO:0022414	Reproductive process	1.70E-50	7.50E-11	1.90E-07	6.30E-38
GO:0050896	Response to stimulus	6.00E-31	5.50E-06	8.80E-07	8.60E-44

## Discussion

A large number of variants were found significantly associated with milk, fat and protein yield. Our findings support previous QTL findings from the Nordic Red breeds, e.g. on BTA 5, 14, 20 [37, 38] and locate new variations that are good candidates to be causative variations. This is the first time when NRC population is studied with imputed whole genome sequence variants in order to refine QTL associated with milk production. However, it is still difficult to pinpoint the causative variant among several closely linked, almost equally significantly associated variations. One way to classify the variations is to look at the predicted functional consequence of the SNP [29]. The possibility that a variation has an impact on the phenotype is higher if the variation causes an amino acid change (missense variation) which is predicted (e.g. with SIFT analysis, [29]) to have an effect for protein function, is located on splicing site, or is located downstream or upstream of the known gene (possible regulatory regions of the transcription). On the other hand, genome annotation for cattle is still incomplete and most regulatory elements remain unknown. In the search for biologically relevant markers the information of interactions between genes in known pathways or networks can be useful. In this study, we identified some interesting gene interaction networks based on the significantly associated variants within genes (even though the functional effects of the variants could not be predicted). The results may be used to have a closer look at also other genes in the indicated networks for functional variants.

Although no common SNPs were found associated with milk production traits and fertility, the five common genes between fat yield and fertility give some indication of the relationship between those traits. The genes *bta-mir-146b* and *GFB1* are associated with a fertility gene network linked with inflammatory response and cell-to-cell signaling and the fat yield network connected with lipid and carbohydrate metabolism. Further support was gained from the gene enrichment analysis, both the traits show significant enrichment of the genes related for example to reproduction and reproduction and reproductive processes, altogether having 16 common GO terms.

From the four chromosomes reported to harbor highest number of QTL for milk production [39], two were indicated by our data (BTA14 and BTA20). Strucken et al. [40] summarized 14 genes from ten different chromosomes to be the major genes involved in milk production. Among those genes are *DGAT1* and *GHR.* Some commonly found QTL (e.g. BTA6, [41]) were not seen in our data; that could be due to fixation of the QTL or very low MAF in the NRC population. One explanation could be that the EMMAX method chosen for association analysis might be too conservative. EMMAX uses approximations for constructing test of the fixed SNP effects of interest in the linear mixed model because fitting a full linear mixed model for each SNP in turn across the genome is computationally challenging [42]. This leads to systematic underestimation of the most significant *p* values [43], but makes EMMAX one of the fastest LMM based programs [42]; an argument that has to be considered when having whole genome sequence level data from substantially large amount of individuals.

### *DGAT1* (BTA14)

The strongest signal for association was found from BTA14 for fat and milk yield. Also protein yield is significantly associated with the same region on BTA14. The top SNP varied depending on the trait (see Table 2). Daetwyler et al. [13] analyzed association of early lactation milk fat percentage with whole genome sequence variation data in Fleckvieh and Holstein bulls. As in our study, the previously reported causative variation *K232A* (bp 1,802,266, [35]) of *DGAT1* gene was not the variant with the lowest *p* value in Holstein and Fleckvieh for milk fat production although *K232A* was among the top SNPs. Pausch et al. [44] used whole genome sequence data to impute German Fleckvieh and Holstein-Friesian cattle genotypes from a larger set of animals candidate regions and were able to confirm the association of *K232A* mutation, however, their approach was biased as only known candidate SNPs were tested for association.

In our data, closer inspection of the associated variations (−log_10_(*p*) ≥ 8.50, fat yield) in the *DGAT1* region revealed that when the effect of the *K232A* mutation was fixed, no additional statistically significant SNP effects were left. The causative mutation (*K232A*/rs109326954) of *DGAT1* was reported already over a decade ago [45] and has been functionally confirmed [35]. The K allele increases milk fat percentage [35], whereas allele A increases milk production [46]. There are different possible explanations why *K232A* did not turn out to be the most significantly associated variation in this study. Imperfect imputation may affect association results. Accuracy of the imputation is considerably improved by increasing the size of reference panel, i.e. sequenced animals [47] and imputation accuracy seems to be higher when populations under study are combined for the imputation processes [13]. Our reference panel consisted of a multi-breed population with 1228 individuals from several breeds including both dairy and beef cattle. The *DGAT1* region would be an interesting candidate to study with the information from 1000 Bulls Genomes Project [13, 21]. It would give a chance to study the haplotype structure of the region in the cattle population worldwide and possibly trace back the evolution of the QTL effect.

### *MGST1* and *MKL1* (BTA5)

Viitala et al. [16] showed that Finnish Ayrshire has a milk production QTL at the proximal end of BTA5. Wang et al. [48] reported of a QTL for milk fat percentage in German Holstein-Friesian at the location of 94,551,792 bp and suggested a candidate gene to be *EPS8*. One of the most significant SNP in the study by Aliloo et al. [11] for milk yield (Jersey and Holstein) was located at bp 94,518,850 on BTA5. For fat yield, we observed an association peak at bp 93,945,694 (Table 2). This variation is in intronic region of the gene *MGST1* having a role in oxidative stress reaction [49] QTL peak for the milk yield on BTA5 is located within an intron of the *MKL1*gene (bp 112,343,204). *MKL1* is related to transcription regulation. Protein yield association peak is located at bp 112,450,860 on BTA5 and is annotated to the non-coding region. Previously QTL related to body weight have been mapped nearby [50].

### *GHR* and *PRLR* (BTA20)

BTA20 is among the chromosomes harboring many QTL related to milk production [39]. *GHR* has been reported as one of the major genes involved in milk production [40]. First Blott et al. [36] found that variation *F279Y* (bp 31,909,478) in the *GHR* gene is associated with a strong effect on milk yield and composition and other studies have confirmed it (e.g. [44]). Other variations than *F279Y* have also been found to affect milk production nearby the *GHR* gene [38]. In our study, *F279Y* has the strongest association to milk yield among the NRC population on BTA20. Further confirmation for the causality of the *F279Y* comes from SIFT analysis predicting the mutation to be deleterious for protein structure, i.e. potentially altering the protein structure thus possible leading to changes in function of the protein. The top SNP (bp 31,909,478) in the *GHR* gene region is clearly the most significant one (Additional file 10) in contrast to *DGAT1* region where several variations are strongly associated to milk yield (Additional file 7). The Y allele is predicted to be unfavorable for milk production [36], but it is still fairly common in the NRC population. *GHR* has been suggested to be under balancing selection because of the observed high variation in the cytoplasmic region [51].

Another gene on BTA20 of special interest is *PRLR* and the variation S18N (positions 39,115,344-39,115,345) [37]. However, it has been suggested that S18N is rather linked to the causative mutation than being causative itself [44]. We found that the variation at bp 38,828,254 on BTA20 located in the intergenic region, was indicated to be the most likely candidate responsible for the QTL effect seen. This variation is located approximately 245,000 base pairs downstream of the *PRLR* gene and additional studies are required to resolve the mechanisms how it may influence milk production.

### *TRIM26* (BTA23)

An intronic variant in the gene *TRIM26* at bp 28,567,796 on BTA23 has an association with fat yield. The function of the *TRIM26* gene, a member of the tripartite motif (TRIM) gene family, is unknown [52]. It is located close to the major histocompatibility complex (*MHC*) class I region. Feed intake QTL have been mapped close to the association peak observed in this study [53].

### *PAM16*, *UNKL* and *CLEC16A* (BTA25)

Altogether six association peaks (QTL) were observed from BTA25 for different traits. The same variation (bp 3,498,960) in gene *PAM16* is associated with both milk and protein yield. The gene has a critical role in protein translocation across the inner mitochondrial membrane [54]. Other QTL on BTA25 were found for milk yield in the intergenic region (bp 2,669,704) and for protein yield QTL at bp 1,103,856 in the gene *UNKL* that has a role on protein, zink ion and metal ion binding. Two distinct QTL were identified for fat yield, peak variations located at positions 9,870,005 and 36,226,978. Variation at bp 9,870,005 is located in the *CLEC16A* gene (Table 2). Variations of *CLEC16A* in humans are associated with increased type I diabetes risk [55]. In addition, milk protein percentage QTL has previously been found from the region 9.3–10.6 Mb [56].

### *NEURL1* (BTA26)

After the significance test by fixing of the top SNPs, two QTL were left on BTA26 for fat yield at bp 24,379,571 in *NEURL1* gene and at bp 44,802,991 (Table 2). *NEURL1* gene is associated with lactation (GO term 0007595) thus making the variation an interesting candidate to be a causative mutation.

## Conclusions

Association analyses among Nordic Red Cattle using over 15 million sequence variations across the whole genome imputed for over 4000 progeny tested Nordic Red Cattle bulls indicated several variations likely to have an impact for milk production. We show that imputation is robust and cost-effective way to expand the information available and to increase knowledge of the causative mutations affecting traits important to production animals. The availability of the whole genome level sequence data opens endless possibilities to study quantitative trait architecture more closely. Still finding the quantitative trait nucleotides is challenging, with linkage disequilibrium and many small-effect QTL creating the puzzle that is not easy to solve. Furthermore, better annotation of the cattle genome is required to be able to predict the effects of variations on the phenotypes more accurately. The knowledge from gene interactions (although human/rodent based) may help to identify likely candidate genes and variations. Network and pathway information also indicates ways through which the traits are correlated.

## Availability of data and materials

Genome assembly data were taken from publicly available sources. The assembly is available for download (ftp://ftp.ncbi.nlm.nih.gov/genomes/Bos_taurus/GFF/). Part of the whole genome sequencing data from the 1000 Bull Genomes Project are publically available (variations in dbSNP (http://www.ncbi.nlm.nih.gov/projects/SNP/) and sequence data at NCBI using SRA no. SRP039339 (http://www.ncbi.nlm.nih.gov/bioproject/PRJNA238491)) and for the rest, the Board of the 1000 Bull Genome Consortium should be contacted. All annotation information was obtained from a publicly available source (http://www.ensembl.org). Whole genome sequences from Aarhus University and individual SNP genotype data is available only upon agreement with the breeding organization and should be requested directly from the authors.

## Ethics (and consent to participate)

Not applicable.

## Consent to publish

Not applicable.

## Additional files

Additional file 1: List of variations tested by fixing the effect of the variation in question. (TXT 226 bytes) Additional file 2: SNPs with Bonferroni corrected *p*-values higher than 0.05 (−log_10_(*p*) ≥ 8.50) for the fat yield. Columns on table: SNP = marker name, CHR, position on bp, alleles, Beta = variation effect, SE = standard error for effect, P = *p*-value, minuslog10 = (−log_10_(*p*)-values), Bonferroni corrected *p*-value. For the rest of the columns, see explanation from http://www.ensembl.org/common/Help/Glossary?db=core. (XLSX 859 kb) Additional file 3: SNPs with Bonferroni corrected *p*-values higher than 0.05 (−log_10_(*p*) ≥ 8.50) for the milk yield. Columns on table: SNP = marker name, CHR, position on bp, alleles, Beta = variation effect, SE = standard error for effect, P = *p*-value, minuslog10 = (−log_10_(*p*)-values), Bonferroni corrected *p*-value. For the rest of the columns, see explanation from http://www.ensembl.org/common/Help/Glossary?db=core. (XLSX 209 kb) Additional file 4: SNPs with Bonferroni corrected *p*-values higher than 0.05 (−log_10_(*p*) ≥ 8.50) for the protein yield. Columns on table: SNP = marker name, CHR, position on bp, alleles, Beta = variation effect, SE = standard error for effect, P = *p*-value, minuslog10 = (−log_10_(*p*)-values), Bonferroni corrected *p*-value. For the rest of the columns, see explanation from http://www.ensembl.org/common/Help/Glossary?db=core. (XLSX 30 kb) Additional file 5: Significantly associated missense variation listed per trait. (XLSX 26 kb) Additional file 6: BTA5, −log10(*p*) values plotted against the genomic positions for each trait. (PNG 306 kb) Additional file 7: BTA14, −log10(*p*) values plotted against the genomic positions for each trait. (PNG 204 kb) Additional file 8: BTA16, −log10(*p*) values plotted against the genomic positions for each trait. (PNG 415 kb) Additional file 9 BTA19, −log10(*p*) values plotted against the genomic positions for each trait. (PNG 456 kb) Additional file 10: BTA20, −log10(*p*) values plotted against the genomic positions for each trait. (PNG 396 kb) Additional file 11: BTA23, −log10(*p*) values plotted against the genomic positions for each trait. (PNG 519 kb) Additional file 12: BTA25, −log10(*p*) values plotted against the genomic positions for each trait. (PNG 473 kb) Additional file 13: BTA26, −log10(*p*) values plotted against the genomic positions for each trait. (PNG 523 kb) Additional file 14: Plot of the –log_10_(*p*) values on BTA14 for fat yield when causative *DGAT1* variation is fixed. No significance associations left. (PNG 35 kb) Additional file 15: Plot of the –log_10_(*p*) values on BTA14 for milk yield when causative *DGAT1* variation is fixed. No significance associations left. (PNG 36 kb) Additional file 16: Gene networks generated by the IPA® platform for fat yield (a) and fertility index (b). Genes marked with blue are having variations associated statistically significantly. Yellow color represents genes that are having a candidate causative variation for fat yield; genes marked with orange are the ones that have significantly associated SNPs between fertility and fat yield. Genes with white or grey color are added by IPA to connect the network. Dotted lines indicate indirect interactions and solid lines indicated direct interaction between the genes. (PNG 233 kb) Additional file 17: Gene network generated by the IPA® platform for milk yield. Genes marked with blue are having variations associated statistically significantly. Yellow color represents genes that are having a candidate causative variation for milk yield. Genes with white or grey color are added by IPA to connect the network. Dotted lines indicate indirect interactions and solid lines indicated direct interaction between the genes. (PNG 153 kb) Additional file 18: Gene network generated by the IPA® platform for protein yield. Genes marked with blue are having variations associated statistically significantly. Yellow color represents genes that are having a candidate causative variation for protein yield. Genes with white or grey color are added by IPA to connect the network. Dotted lines indicate indirect interactions and solid lines indicated direct interaction between the genes. (PNG 118 kb)
