# Supplementary figures and images for: Genome-wide association analysis of milk yield traits in Nordic Red Cattle using imputed whole genome sequence variants
Source: BMC Genet. 2016 Mar 22;17:55. doi: 10.1186/s12863-016-0363-8 (PMC4804490; doi:10.1186/s12863-016-0363-8)

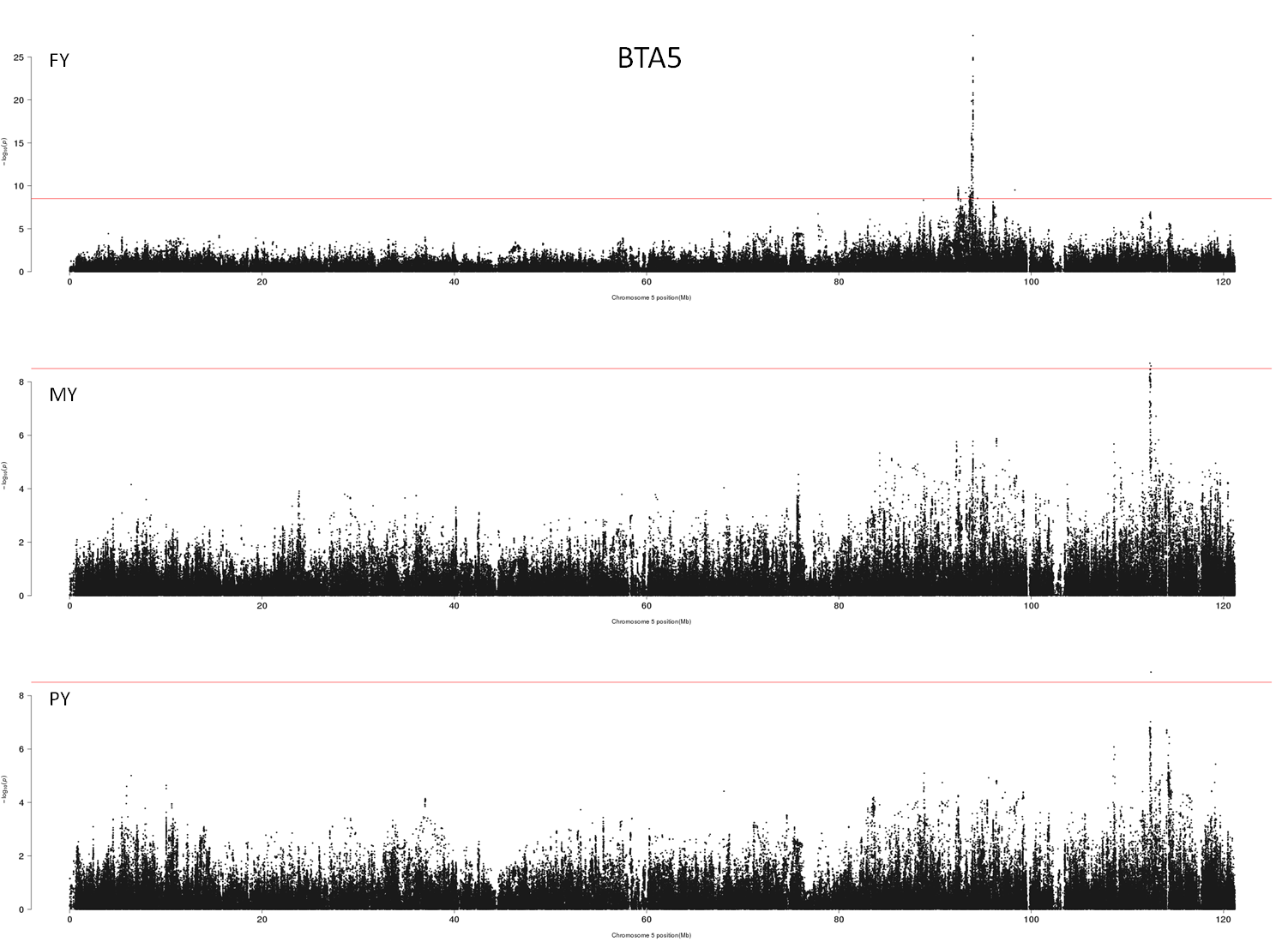

Supplement: Additional file 6: — BTA5, −log10(p) values plotted against the genomic positions for each trait. (PNG 306 kb) [file 12863_2016_363_MOESM6_ESM.png]

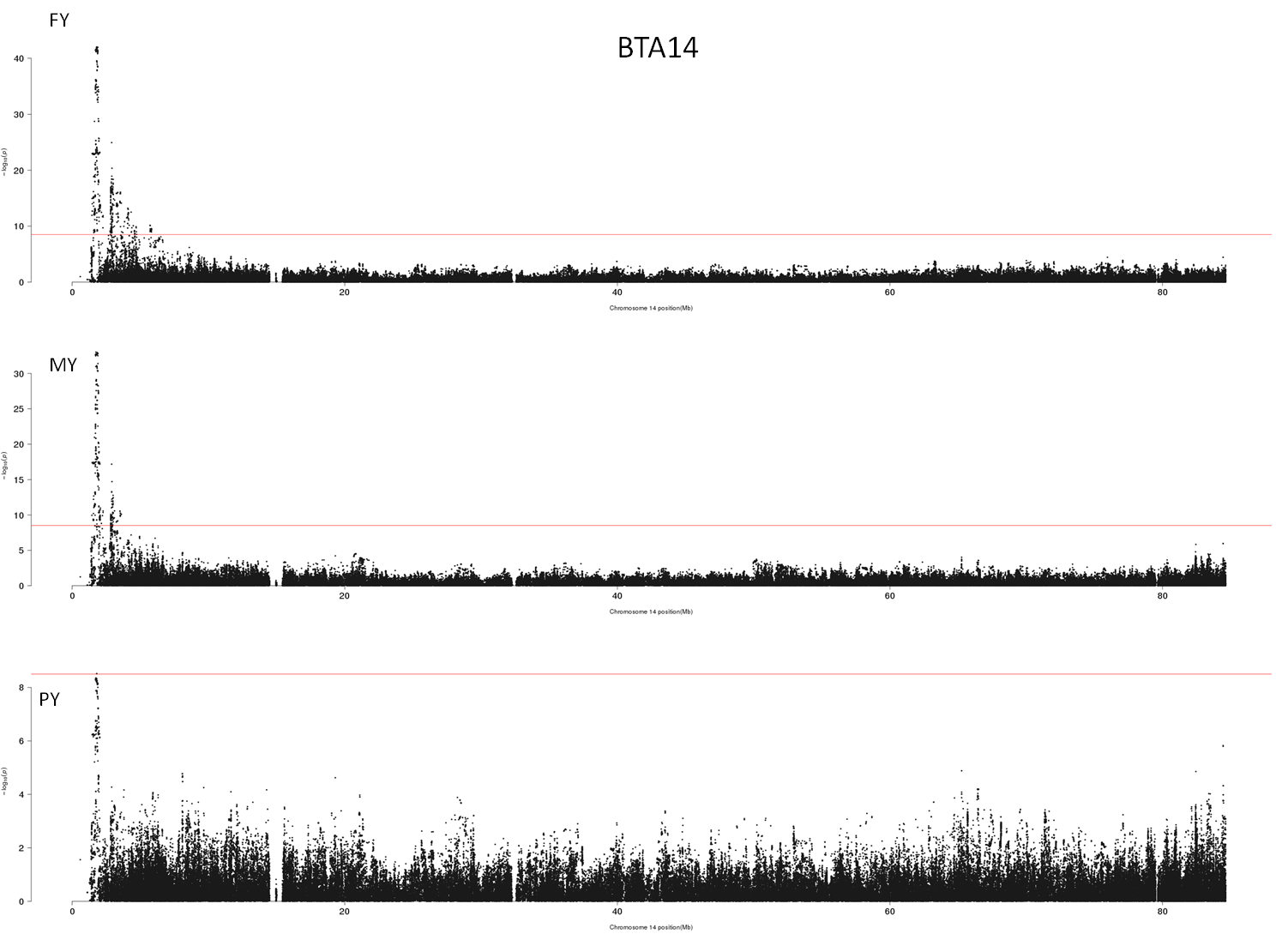

Supplement: Additional file 7: — BTA14, −log10(p) values plotted against the genomic positions for each trait. (PNG 204 kb) [file 12863_2016_363_MOESM7_ESM.png]

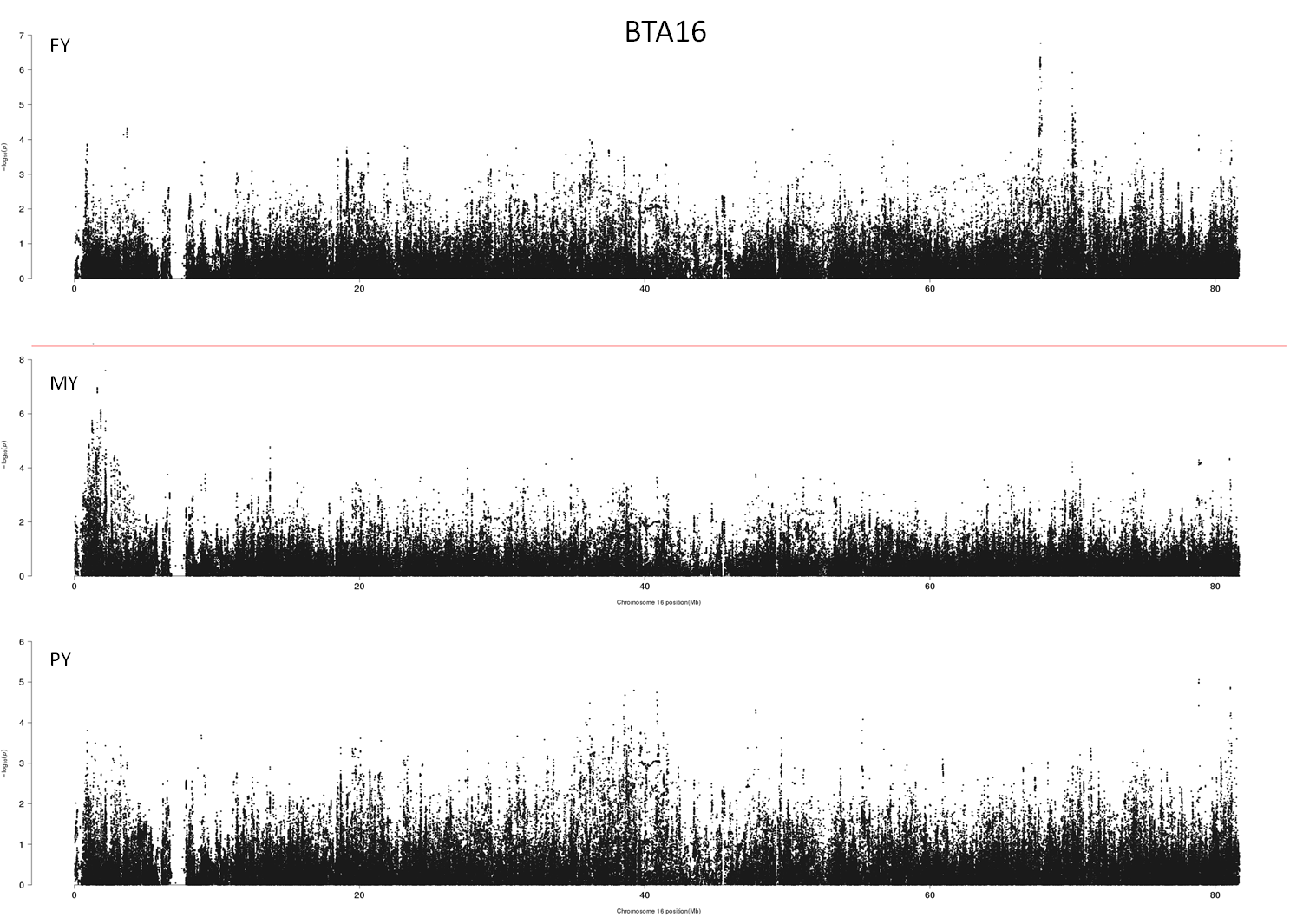

Supplement: Additional file 8: — BTA16, −log10(p) values plotted against the genomic positions for each trait. (PNG 415 kb) [file 12863_2016_363_MOESM8_ESM.png]

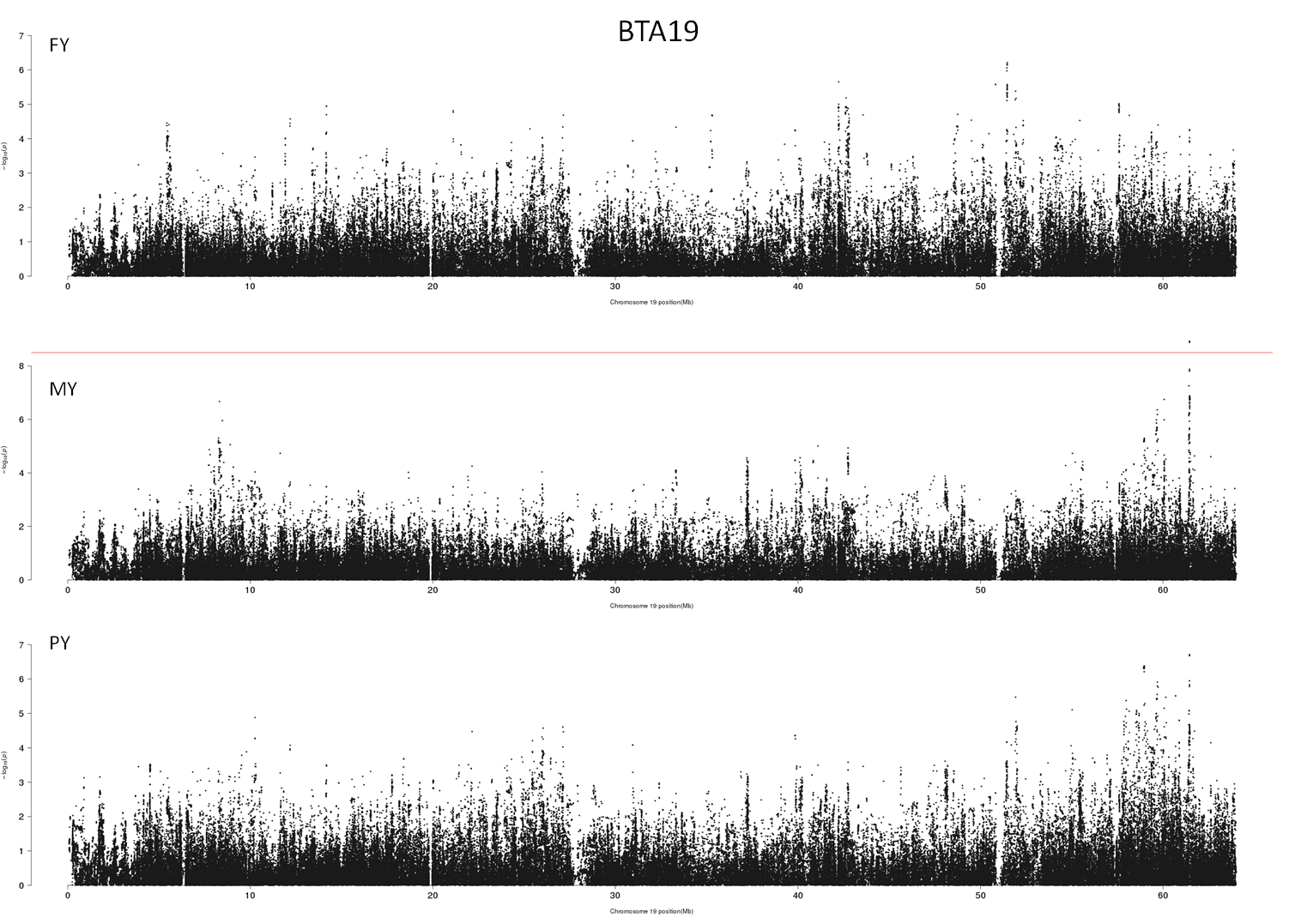

Supplement: Additional file 9 — BTA19, −log10(p) values plotted against the genomic positions for each trait. (PNG 456 kb) [file 12863_2016_363_MOESM9_ESM.png]

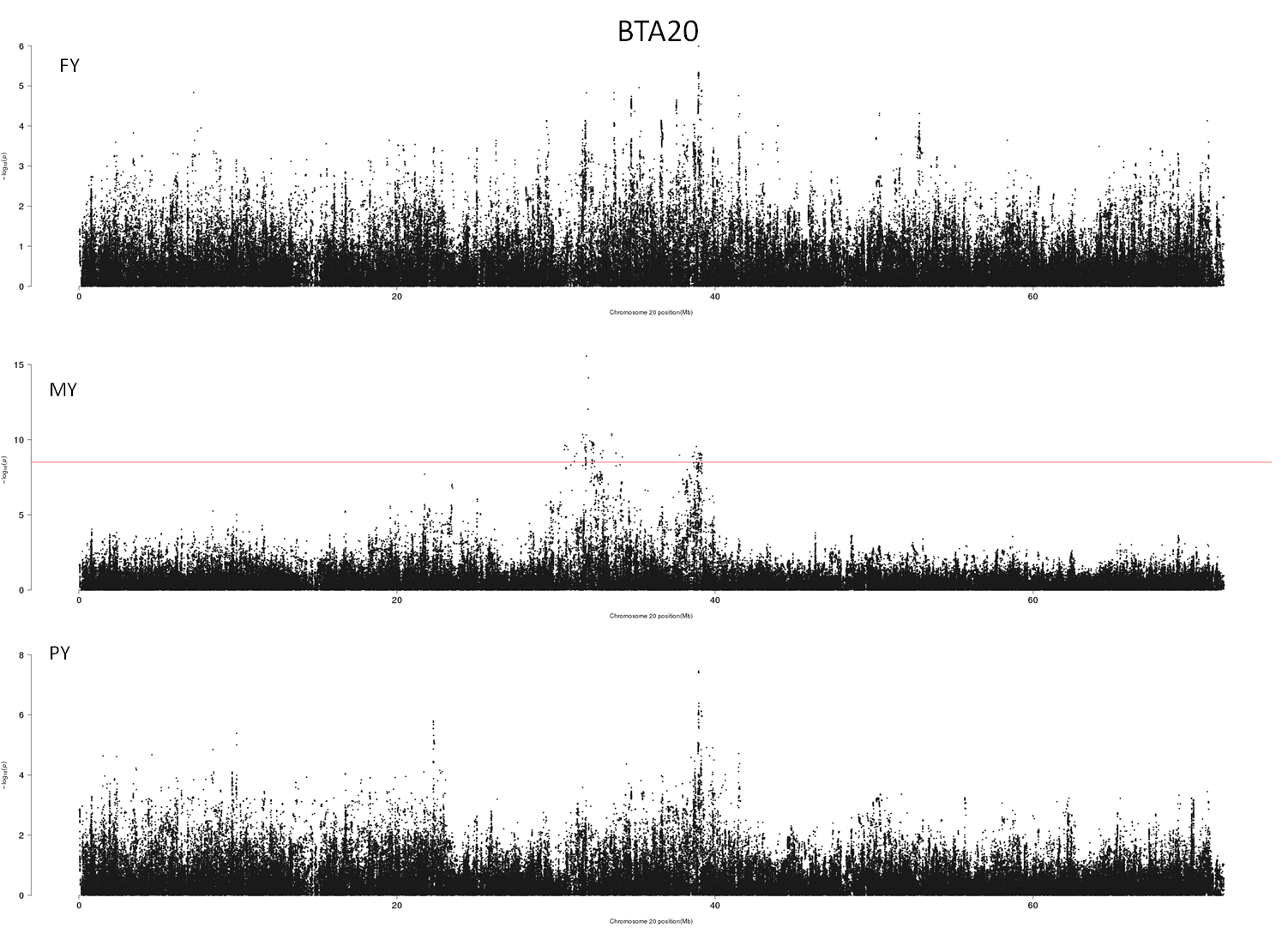

Supplement: Additional file 10: — BTA20, −log10(p) values plotted against the genomic positions for each trait. (PNG 396 kb) [file 12863_2016_363_MOESM10_ESM.png]

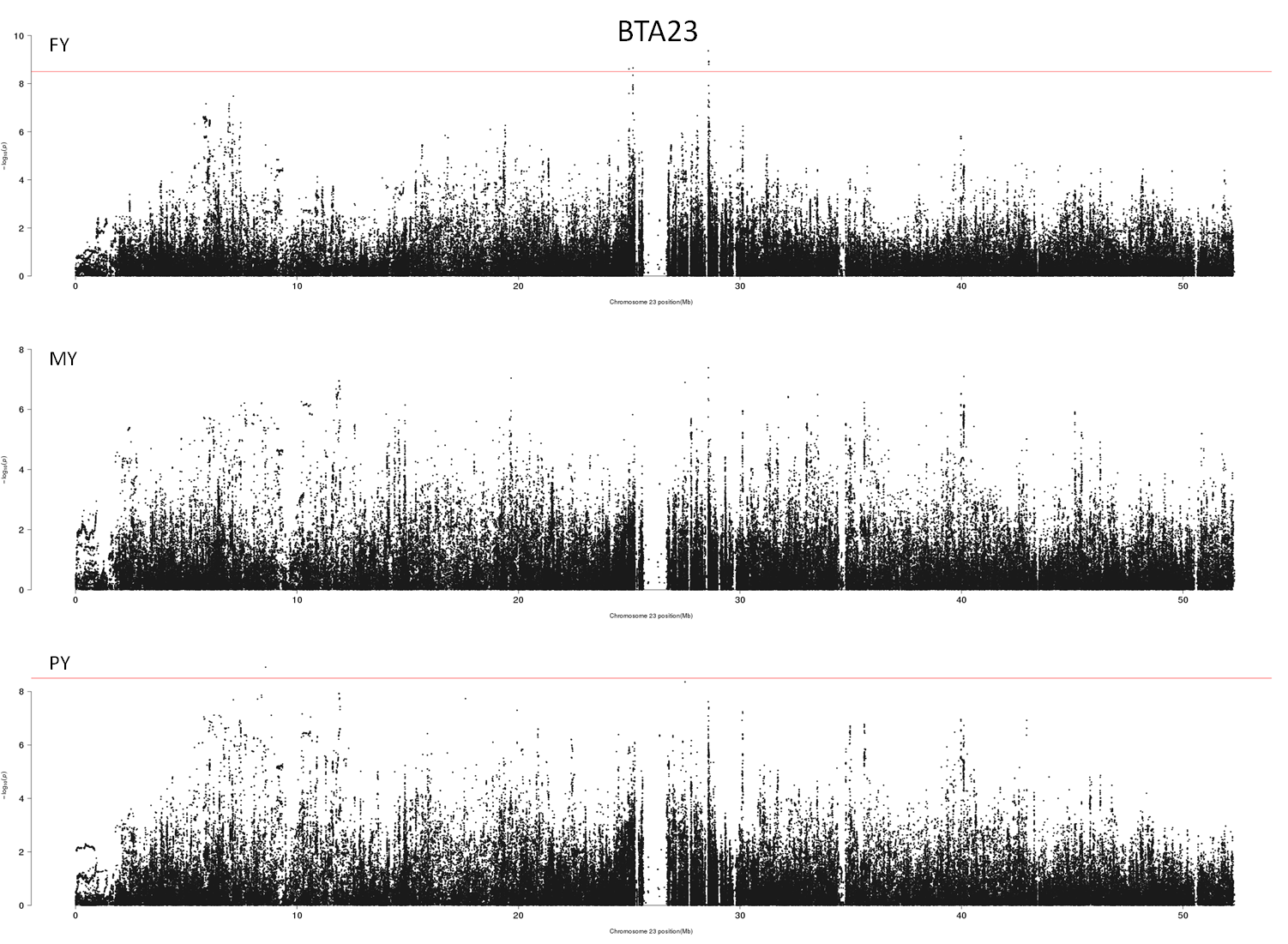

Supplement: Additional file 11: — BTA23, −log10(p) values plotted against the genomic positions for each trait. (PNG 519 kb) [file 12863_2016_363_MOESM11_ESM.png]

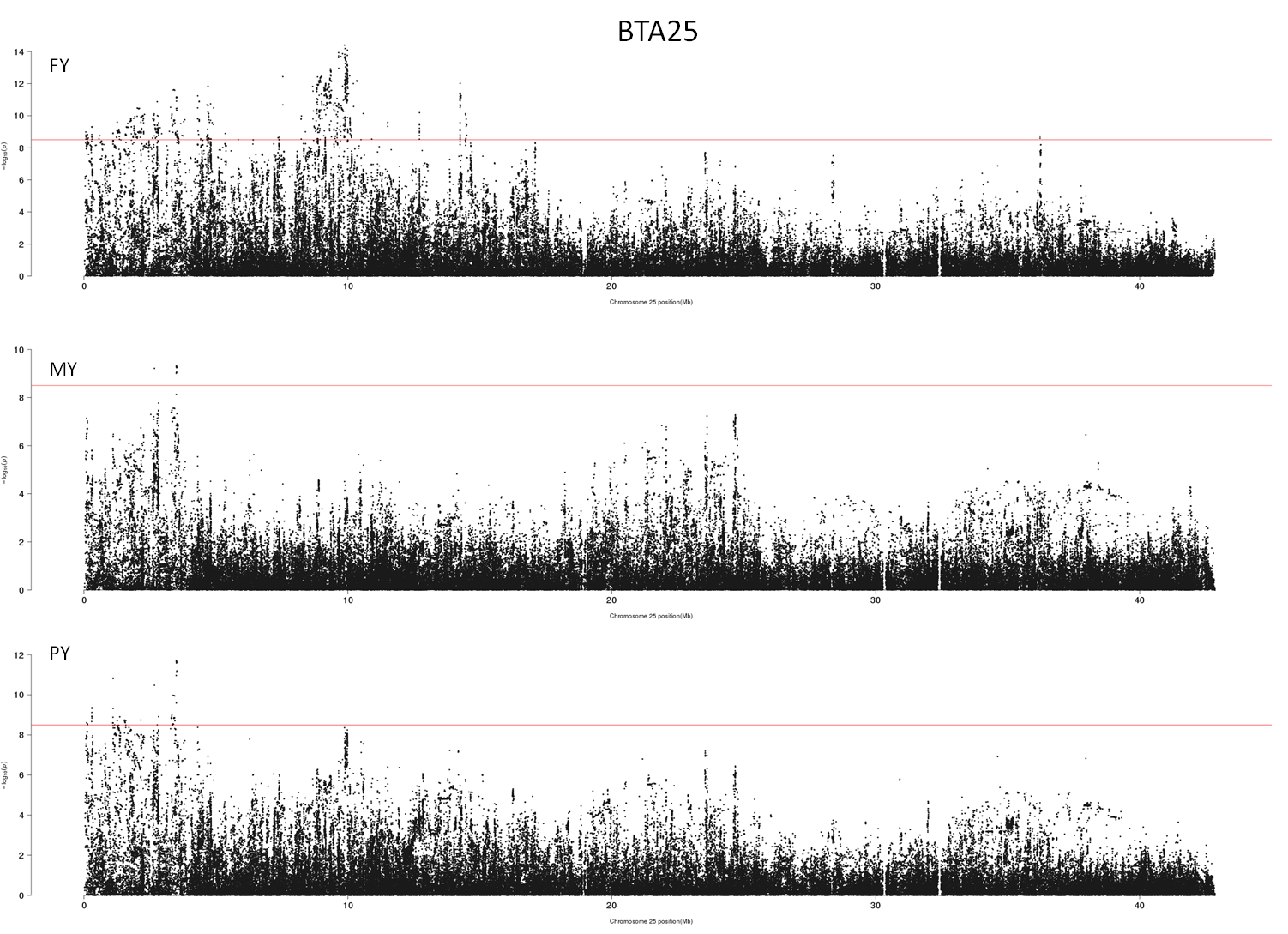

Supplement: Additional file 12: — BTA25, −log10(p) values plotted against the genomic positions for each trait. (PNG 473 kb) [file 12863_2016_363_MOESM12_ESM.png]

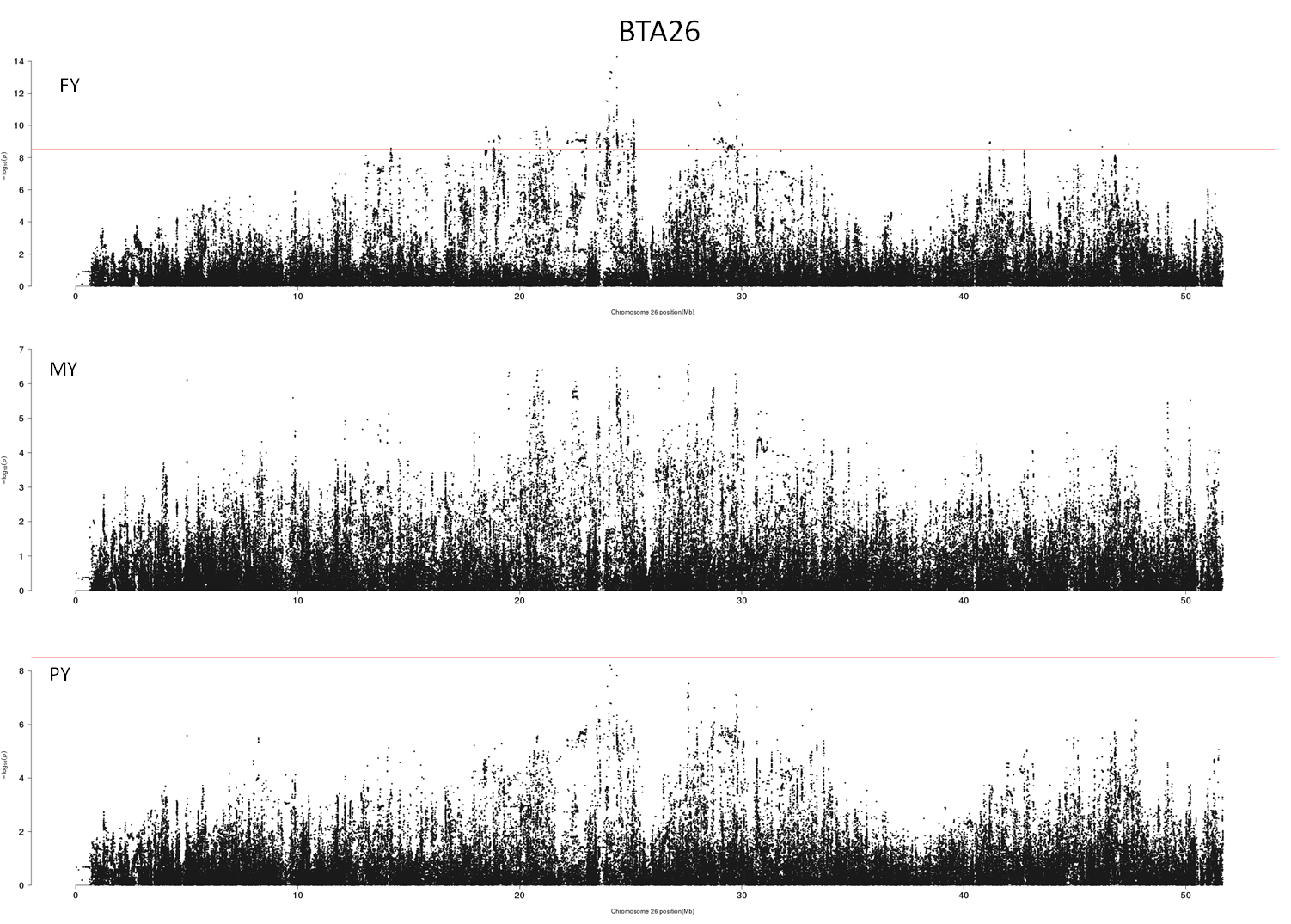

Supplement: Additional file 13: — BTA26, −log10(p) values plotted against the genomic positions for each trait. (PNG 523 kb) [file 12863_2016_363_MOESM13_ESM.png]

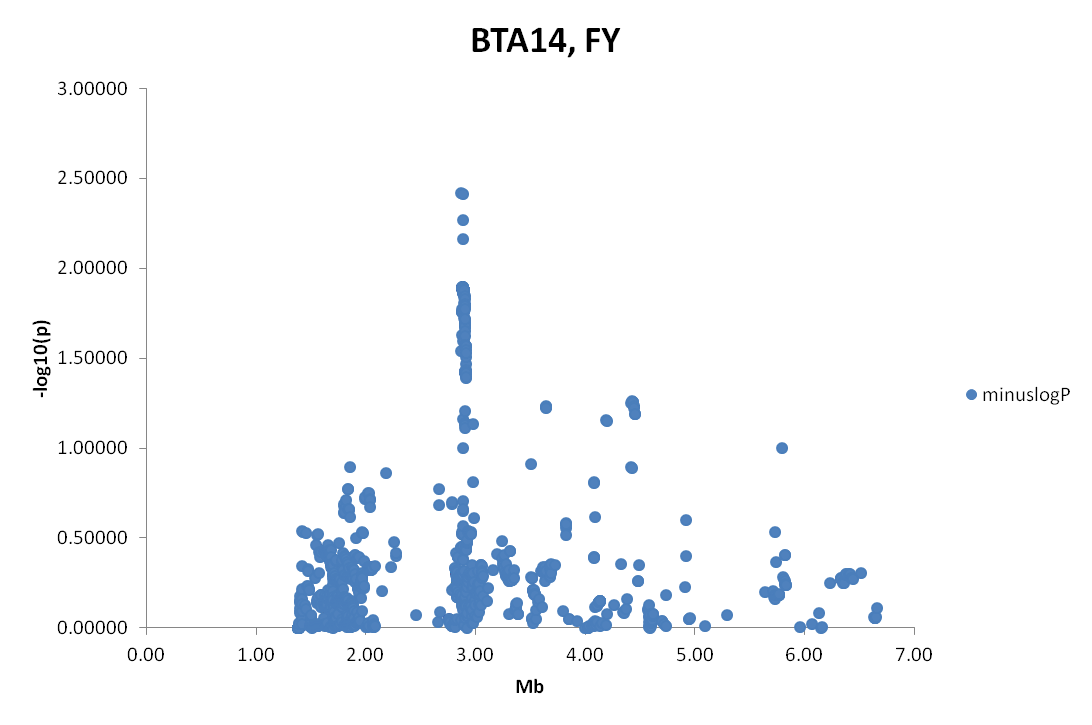

Supplement: Additional file 14: — Plot of the –log10(p) values on BTA14 for fat yield when causative DGAT1 variation is fixed. No significance associations left. (PNG 35 kb) [file 12863_2016_363_MOESM14_ESM.png]

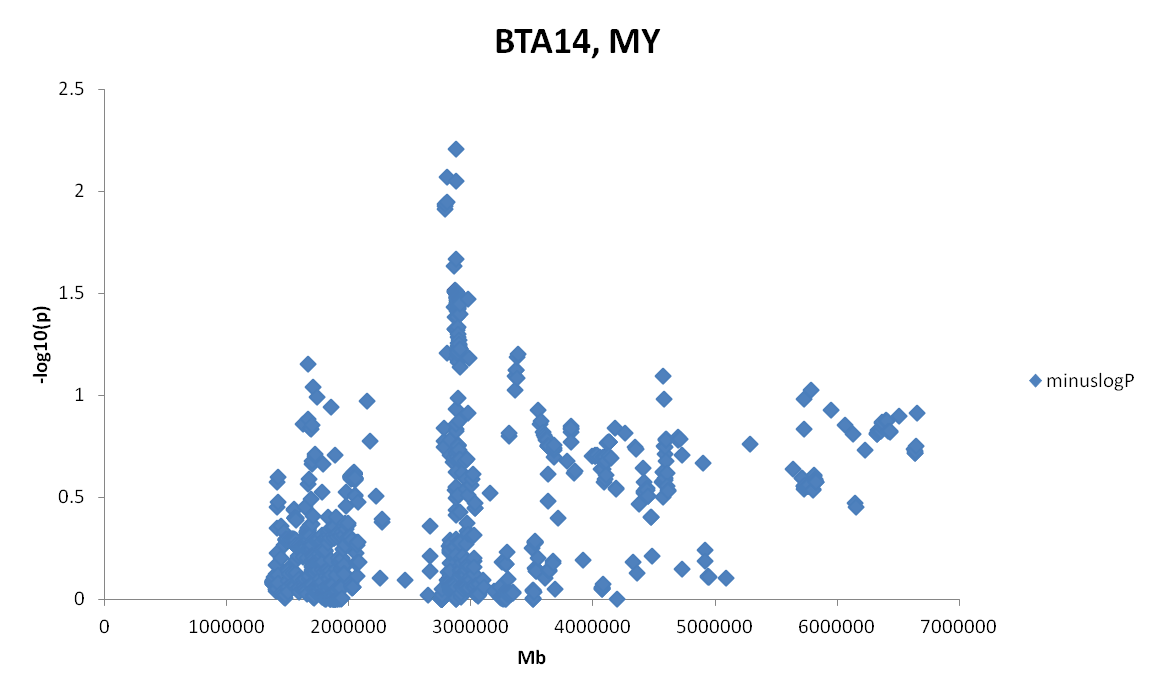

Supplement: Additional file 15: — Plot of the –log10(p) values on BTA14 for milk yield when causative DGAT1 variation is fixed. No significance associations left. (PNG 36 kb) [file 12863_2016_363_MOESM15_ESM.png]

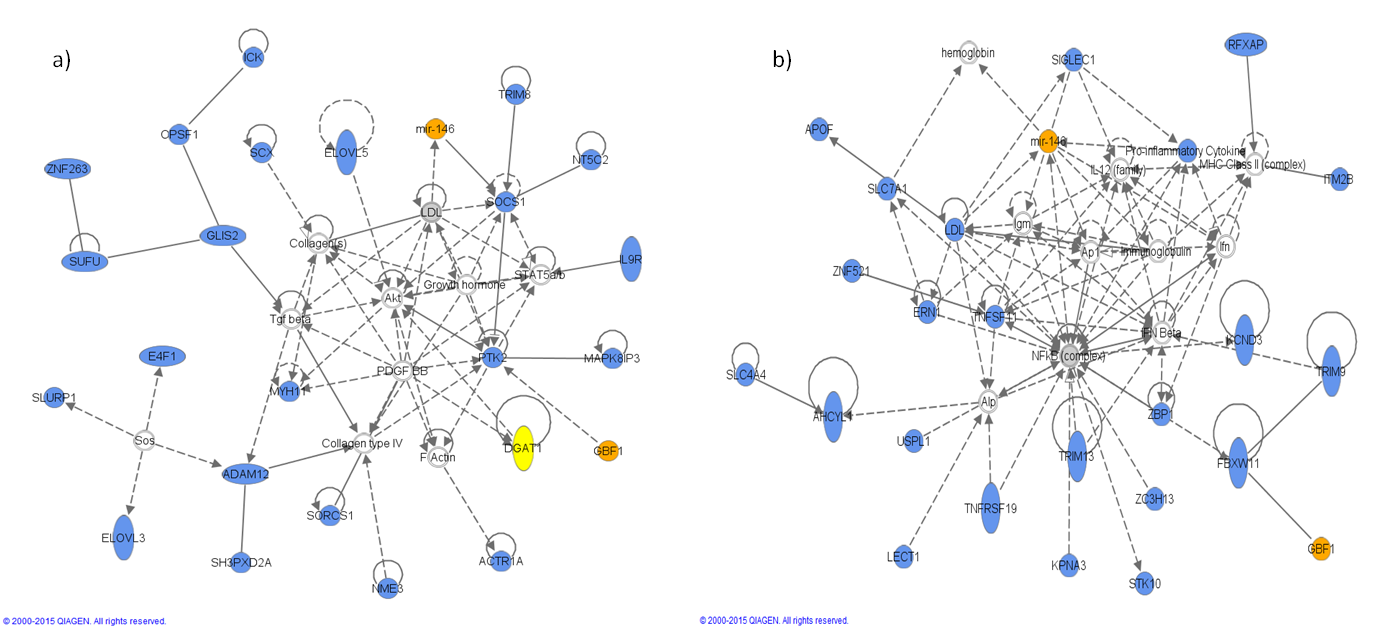

Supplement: Additional file 16: — Gene networks generated by the IPA® platform for fat yield (a) and fertility index (b). Genes marked with blue are having variations associated statistically significantly. Yellow color represents genes that are having a candidate causative variation for fat yield; genes marked with orange are the ones that have significantly associated SNPs between fertility and fat yield. Genes with white or grey color are added by IPA to connect the network. Dotted lines indicate indirect interactions and solid lines indicated direct interaction between the genes. (PNG 233 kb) [file 12863_2016_363_MOESM16_ESM.png]

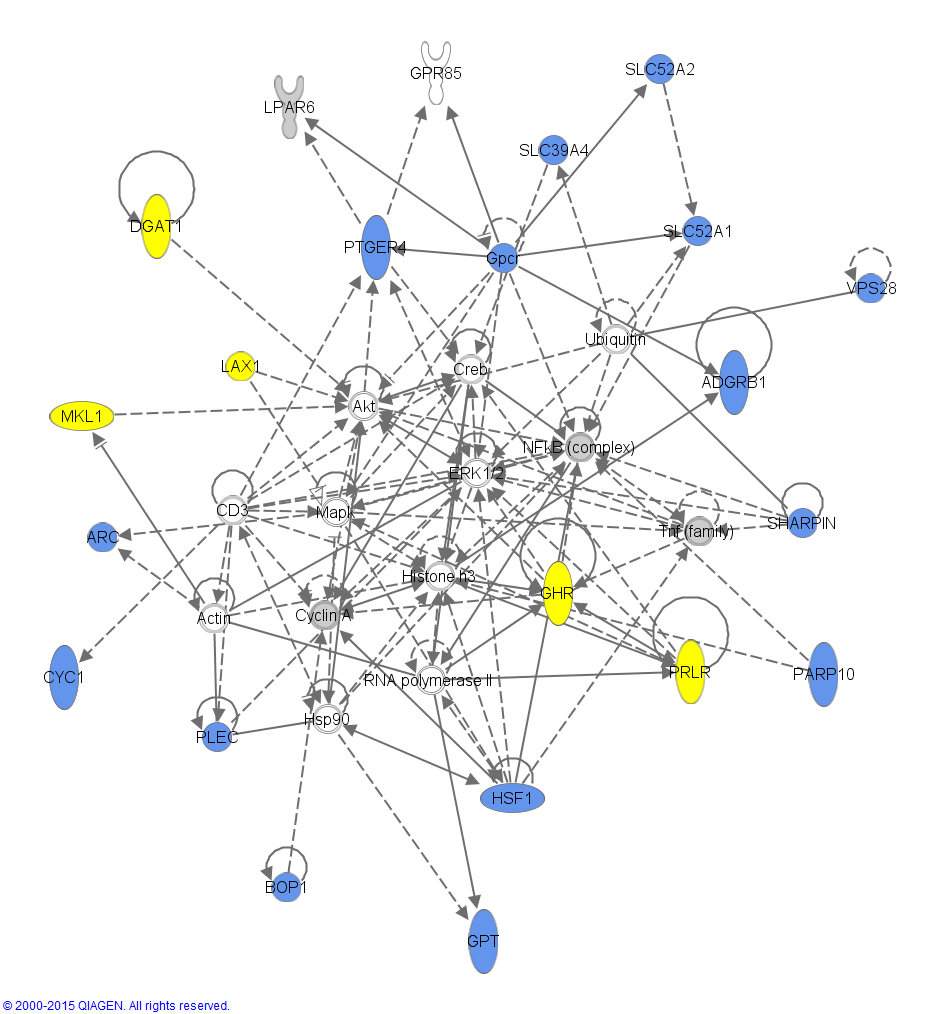

Supplement: Additional file 17: — Gene network generated by the IPA® platform for milk yield. Genes marked with blue are having variations associated statistically significantly. Yellow color represents genes that are having a candidate causative variation for milk yield. Genes with white or grey color are added by IPA to connect the network. Dotted lines indicate indirect interactions and solid lines indicated direct interaction between the genes. (PNG 153 kb) [file 12863_2016_363_MOESM17_ESM.png]

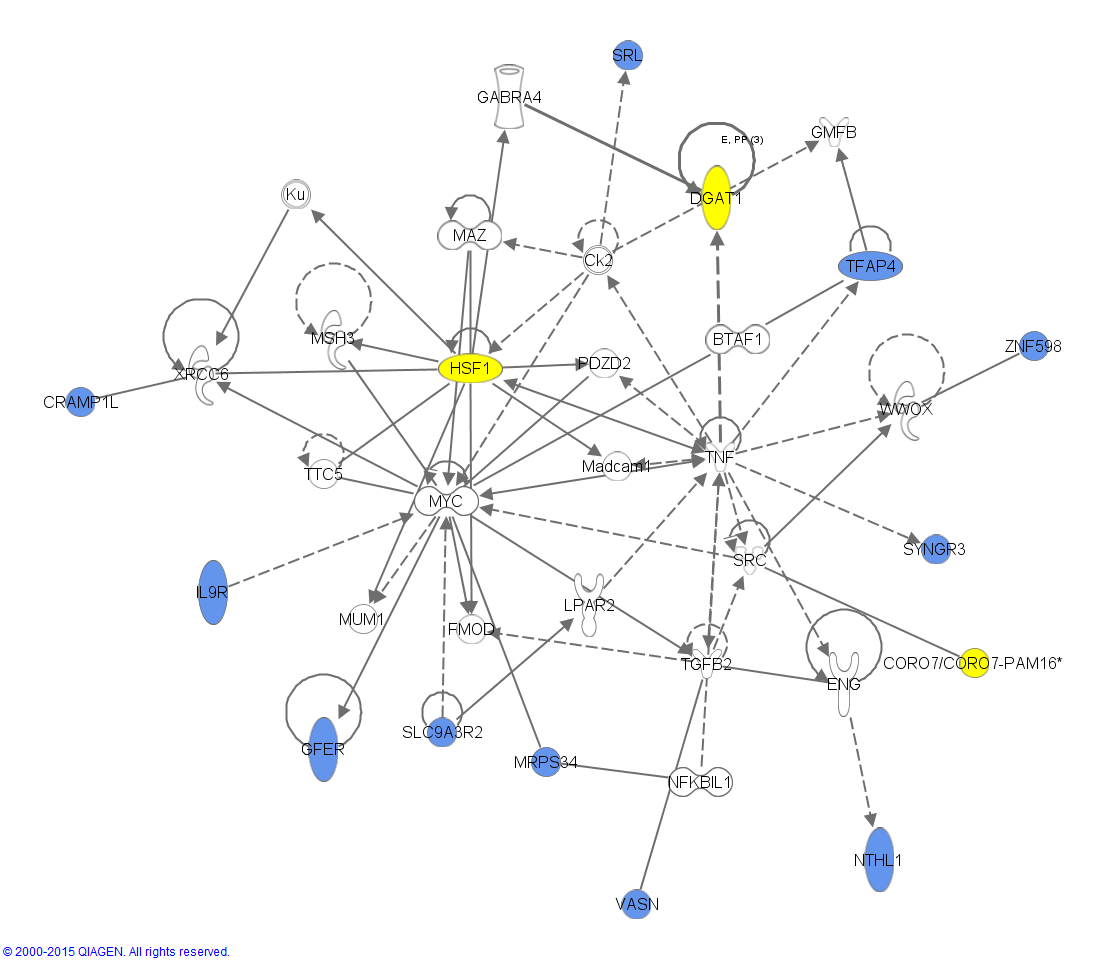

Supplement: Additional file 18: — Gene network generated by the IPA® platform for protein yield. Genes marked with blue are having variations associated statistically significantly. Yellow color represents genes that are having a candidate causative variation for protein yield. Genes with white or grey color are added by IPA to connect the network. Dotted lines indicate indirect interactions and solid lines indicated direct interaction between the genes. (PNG 118 kb) [file 12863_2016_363_MOESM18_ESM.png]
